# Use of Advanced Imaging Techniques for the Characterization of Oily Skin

**DOI:** 10.3389/fphys.2019.00254

**Published:** 2019-03-26

**Authors:** Patricia M. B. G. Maia Campos, Maisa O. Melo, Daiane G. Mercurio

**Affiliations:** School of Pharmaceutical Sciences of Ribeirão Preto, University of São Paulo, São Paulo, Brazil

**Keywords:** oily skin, biophysical and skin imaging techniques, acne, confocal microscopy, cosmetics

## Abstract

Excessively oily skin leads to clinical signs that cause discomfort to patients, such as excessive shine, enlarged pores, acne, and an imbalance of the hydrolipidic layer. In this context, a constant demand for the research and development of products that prevent these features, has been noted in the field of cosmetics and dermatology. Thus, the objective of this study is to evaluate the cutaneous characteristics of oily skin due an excessive production of sebum through biophysical and skin imaging techniques. 19 participants with different skin types were selected and the following parameters were evaluated: pore count, determination of the number of sebaceous glands and amount of sebum in infundibulum, determination of cutaneous microrelief, count of comedones, evaluation of epidermis thickness, characterization of the cellular, and comedone size and its characteristics. These evaluations were done through biophysical and skin imaging techniques. The obtained results showed that different regions of the face presented different characteristics related to oiliness, quantity, and the appearance of pores and comedones. The malar region had a lower epidermis thickness and a larger number of large pores. Moreover, in this region excessive sebum production, which can be related to pores, not comedones, was noted. The nose region presented higher sebum content in the infundibulum and lower active sebaceous glands, showing a higher activity of sebaceous production in this region. The chin region presented a positive correlation between the sebum content, roughness parameter and the number of pores and comedones. As different skin properties are related and influence the appearance of undesirable clinical signs, we identified the need for a multifactorial approach for the effective treatment of oily skin. The rational development of multifunctional cosmetic products that promote the control of oily skin, that regulate the keratinization process, improve the microrelief and leads to a better epidermis and dermis structure, will not only improve oily skin conditions but will also allow for the reduction or disappearance of clinical signs that result from excessive oiliness, all of which causes concern and results in a relentless search for cosmetic and dermatological products that address the unaesthetic nature of these conditions.

## Introduction

In addition to its biological and protective function, skin with a healthy appearance is essential for well-being and a good social life, which is the target of application of cosmetic products and dermatological treatments. However, in order to select the correct treatment and suitable raw materials for development of the formulation, it is important to emphasize the importance of a proper characterization of the skin type and the use of products that are compatible with the skin biology, meeting the expected objectives and avoiding undesirable effects.

Among the existing types of skin, oily skin, which is characterized by a high amount of sebum, especially on the face region, requires special care. Oily skin presents clinical signs that cause discomfort to patients, such as excessive brightness, enlarged pores, and acne, which, in addition to the imbalance of the hydrolipidic mantle, have a negative impact in the quality of life of individuals with this type of skin and can persist throughout life (Baldwin, 2002; Yazici et al., 2004; de Melo and Maia Campos, 2018).

Skin oiliness is related to an excessive production of sebum by the sebaceous glands, usually located in hair follicles, which, along with the gland is referred as a pilosebaceous unit (Thiboutot et al., 2008). The lipids secreted by these glands have several functions, such as maintenance of the integrity of the cutaneous lipid barrier, transport of antioxidants to the skin surface, antimicrobial activity and pro-and anti-inflammatory activities (which can cause a delayed skin aging process) and the generation of body odor and pheromones. Although their function is important, cases of hyperseborrhea cause great discomfort and should be treated (Greene et al., 1970; Downing and Strauss, 1982; Zouboulis, 2004; Zouboulis et al., 2014; Byrd et al., 2018).

Functioning of the sebaceous glands is also related to changes in the physiology of hair follicles, that in combination with other factors can lead to the formation of clinical signs that are typical of oily skin, such as enlarged pores, comedones (which are also inflammatory lesions) and acneic inflammatory lesions (papules, pustules, and cysts) (Zouboulis, 2001).

The term “pore” can be defined as a microphotographic feature on the skin surface which corresponds to enlarged openings of the pilosebaceous follicles (Pierard et al., 2000), and may or may not contain comedones. Several factors, endogenous and exogenous, can be related to pore formation, such as genetic predisposition, gender, aging, hormones, sebaceous secretion, acne, comedogenic substances, and chronic exposure to UV radiation. In general, comedogenic adult acne is caused by environmental factors (Uhoda et al., 2005; Capitanio et al., 2010).

Causes of pore formation can be related to the process of hyperkeratinization of the infundibulum, loss of dermis structural components or even alterations in the epidermis architecture, related to the thickness of the epidermis (Murakami et al., 2006). These features are prevalent in many patients with oily skin, since these structures are more frequent and visible on the face.

With regard to acne lesions, which are characterized by non-inflammatory lesions, open and closed comedones by inflammatory lesions, are called papules, pustules, nodules, and cysts, respectively. The closed comedo is characterized by sebum and skin cells blocking the opening of the follicle and appear as small whitish protrusions beneath the surface of the skin, whereas an open comedo is a non-inflammatory lesion filled with excess oil and dead skin cells. In this case, the skin surface is open and the comedo has a dark appearance, with black or brown coloration on the surface (Ramli et al., 2011a).

Furthermore, there is also so called cosmetic acne, which is a concept introduced by Kligman and Mills (1972), that refers to the formation of acne lesions resultant from the use of cosmetic products containing certain comedogenic ingredients that can interfere with hydrolipidic balance, aggravate the follicle obstruction and can form acneic lesions (Humbert, 2002; Draelos and Dinardo, 2006).

Considering that excess sebaceous production impairs homeostasis, causing an imbalance in the skin, there is a constant demand in the cosmetic and dermatological area for research and development of products that prevent these changes and promote the maintenance of skin eudermia. Therefore, it is important to evaluate the characteristics of oily skin to develop products that allow specific skin care.

### Objective

The objective of this study was to evaluate the cutaneous characteristics that result from excessive sebum production, using advanced techniques of skin imaging analysis, as well as to verify the correlation of the different properties in distinct face regions.

## Materials and Methods

### Participant’s Trials

After approval from the ethical committee of the Faculty of Pharmaceutical Sciences (Protocol n°; 234. – CEP/FCFRP) 19 female subjects aged between 18 and 35 years with skin phototype II, III, and IV and with different amounts of pores and comedones in the skin, were selected. All volunteers participating in the study was guided on the objectives and methods of the research, and, after agreeing to participate in the study, signed the Free and Informed Consent Form. Three face regions (nose wings, chin, and malar) were evaluated, with a demarcated area of 0.8 cm^2^ in each region.

### Count of Active Sebaceous Glands and Quantification of Sebum in the Infundibulum

The activity of the sebaceous glands was evaluated using Sebufix^®^ F16 (Courage & Khazaka, Germany). This equipment consists of plastic foils containing a hydrophobic polymer, which when in contact with the skin oil, turns transparent, allowing the evaluation of the number and size of the spots obtained by the excretion of sebum from the infundibulum. In this study, the tape was placed in contact with the studied regions for 20 s. With this parameter, it is possible to obtain the number of active sebaceous glands and the amount of sebum secreted by them (the image analysis is obtained in mm^2^). The image is obtained using the Visioscan^®^ VC 98 camera and analyzed with the SELS (Surface Evaluation of the Living Skin) software (Dobrev, 2007).

### Count of Skin Pores

In order to count the number of pores in the skin, we used Visioface^®^ equipment (Courage & Khazaka, Germany), a system created to obtain high resolution photos of the face, composed of a digital camera with a white light of diode. VisioFace^®^ Quick software allows the evaluation of parameters of interest, such as skin pores and wrinkles on the skin surface. Three images at different angles were obtained for each participant. To classify the pores as fine or large, the obtained image was taken as a base. The pores visible on the image were marked and interpreted as a percentage according to the whole image. Then, the classification of large of fine pores was done according to the obtained percentage in the analyzed image (Courage and Khazaka electronic GmbH, 2014; Martini and Maia Campos, 2018).

### Evaluation of Epidermis Thickness and Characterization of the Size and Cellular Features of Pores and Comedones

The evaluation of epidermal thickness, cellular and tissue characteristics of pores and comedones was performed using the Vivascope^®^ 1500 Reflectance Confocal Microscopy– RCM (Lucid, United States), which allows *in vivo* non-invasive skin evaluation with a resolution similar to conventional histology, without any risk to the patient and which is totally painless.

Macroscopic images were obtained through the digital macroscopic camera (VivaCam^®^) for the count of comedones. Counting was performed using Image J^®^ software. Microscopic images were obtained using two image acquisition systems, the Vivablock, a 5 mm^2^ image representing tissue sections arranged to map a tissue region at a single depth, and Vivastack, which is multiple successive depths of a certain tissue site, 3-by-3 μm and up to 150 μm of depth (Bielfeldt et al., 2011).

### Determination of Skin Microrelief and Count of Comedones

The Visioscan^®^ VC 98 device (Courage & Khazaka, Germany) was used to evaluate the skin microrelief, which evaluates skin surface characteristics using optical profilometry techniques with a camera scanning process. The obtained parameters were related to skin roughness (Rt), texture (SEr), desquamation (Sesc) and smoothness (SEsm) and calculated by SELS software (Surface Evaluation of the Living Skin). The images were used for the comedones count, which is defined by characteristic black spots in the study regions, and for that, images were obtained in triplicate. The counting was performed using Image J^®^ software (De Paepe et al., 2000; Koh et al., 2002).

### Statistical Design

To evaluate the correlation between all the study parameters in each face region, the Pearson correlation was used with the aid of the GraphPad Prism 5 software. To determine significant differences in skin properties for each region, ANOVA test was used. For the comparison of Epidermal thickness, a Student *T*-test was used. A *P*-value < 0.05 was considered as significant.

## Results and Discussion

Oily skin presents clinical signs such as open pores, comedones and inflammatory acne, which have a negative social and emotional impact due to their unaesthetic nature. A detailed clinical evaluation of these characteristics is therefore necessary.

Currently, advanced techniques of image analysis involves highly advanced technology and has been of great importance as a complementary or alternative dermatological clinical evaluation of the characteristics of oily skin (Ramli et al., 2011b). In the present study, the clinical study of standardization techniques of image analysis, characterization and counting of pores and comedones was carried out, allowing for correlations between different characteristics of face regions. Facial skin presents properties that are distinct from the skin on other body parts, with distinct biophysical, morphological, and structural characteristics (Wa and Maibach, 2010).

**FIGURE 1:**
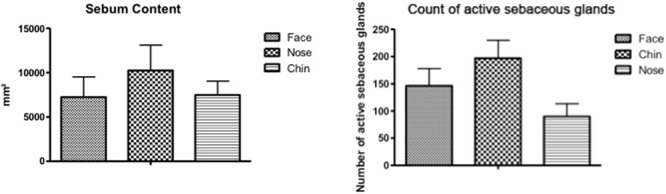
Sebum content in the infundibulum (mm^2^) and count of active sebaceous glands.

**FIGURE 2:**
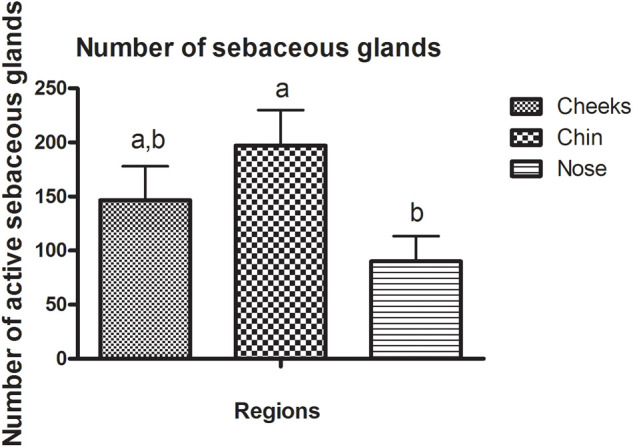
Number of active sebaceous glands in different face regions. Different letters indicate statically different means (*p* < 0.05).

By evaluating the regional characteristics, the results obtained in the present study by the Sebufix^®^ F16 equipment observed a higher production of sebum in the nose region, compared to other regions (Figure 1). However, in relation to the count of active sebaceous glands (Figure 2), the nose region presented a lower count, concluding that, despite the smaller number of glands, this region has a higher activity of sebaceous production.

In a study by Youn et al. (2005), the nose region showed the highest lipid content evaluated by the Sebumeter^®^ equipment. Other studies correlate a lower lipid content of the malar region, compared to T zone regions (forehead, nose, and chin). In addition, when evaluating different parts of the malar region, we observed that regions closer to the nose had higher lipid content (Wa and Maibach, 2010).

A recent study from our research group evaluated the characteristics of oily skin in participants in a higher age group, showing differences compared to a population with dry skin. Beyond the expected differences in the sebum content, it was possible to observe differences in the skin microrelief and echogenicity, confirming that differences persist across all age groups (de Melo and Maia Campos, 2018).

**FIGURE 3:**
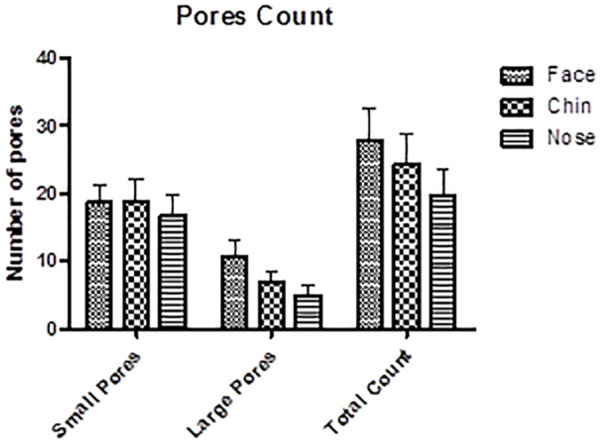
Count of small, large, and total pores in the different study regions.

Pores, which are visible openings on the skin surface, can be related to sebaceous secretion, the process of hyperkeratinization of the hair follicle infundibulum and also have a relation with the epidermis and dermis structure of support. In relation to the number of pores, the chin and the malar region had a higher number of pores in relation to the nose, and the malar region presented a higher number of large pores, evaluated by Visioface Quick^®^ software (Figure 3).

**FIGURE 4:**
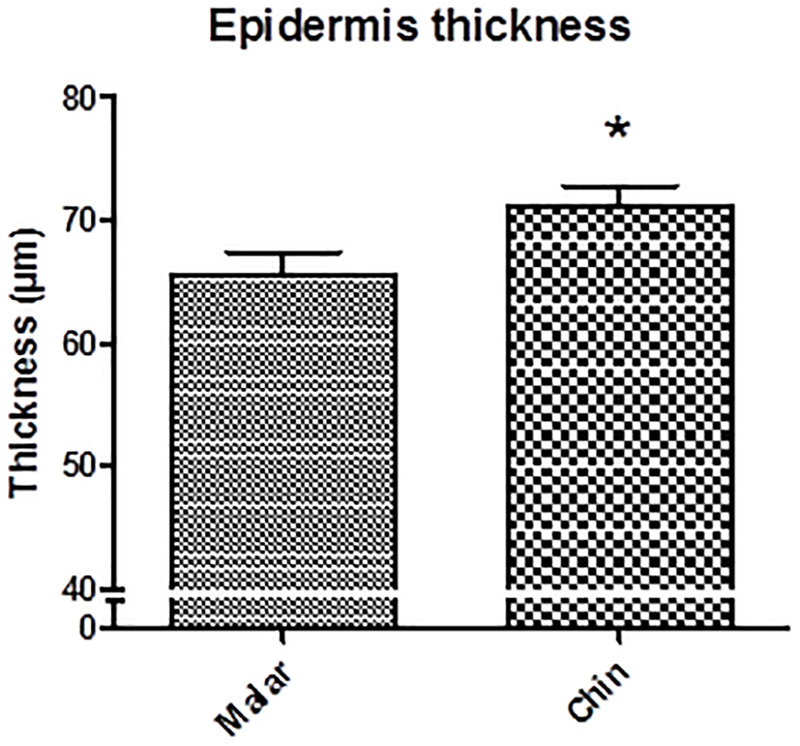
Epidermis thickness in the characterization study. ^∗^Different statistical means as compared the malar region (*p* < 0.05).

Facial regions also present differences in the epidermis and dermis thickness. The nose region presents a greater thickness of these tissues, guaranteeing greater sustentation and, therefore, would not be a region with characteristics favorable for the formation of large pores (Contet-Audonneau et al., 1999; Pellacani and Seidenari, 1999). The greater amount of large pores in the malar region may also be related to structural factors, since the results obtained in the present study (Figure 4) and data reported in the literature, show that the epidermis has lower thickness in this region, which may favor larger pores (Murakami et al., 2006; Andrade et al., 2015). In addition, Kim et al. (2011), evaluated the correlation of different parameters with the presence of pores and observed the relationship between a higher number of pores and lower skin elasticity, showing that a firmer and more structured skin may be related to a lower tendency of pore appearance.

**FIGURE 5:**
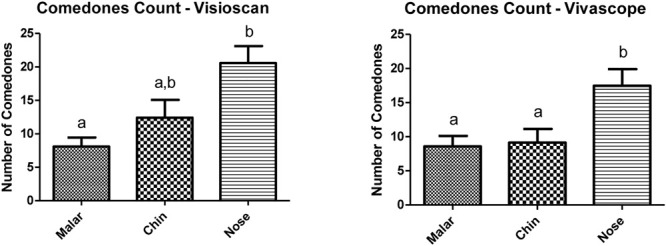
Number of comedones evaluated by the Visioscan^®^ VC 98 and Vivascope^®^ 1500 (macroscopic image) equipment.

Considering that, in the comedone count, the nose region had the largest number, compared to the other study regions using both counting methods (Figure 5). It was also the region that presented the highest content of sebum in the infundibulum (Figure 1), highlighting the relationship between skin oiliness and comedone formation.

The correlation between the results of the studied parameters in different regions is described in Tables 1–3. “+” indicates a positive Pearson correlation (*p* < 0.05).

**Table 1:** Correlation between the evaluated parameters on the malar region.

	Ser	Rt	Seb %	Cont. glan.	Comed. Vivascope	Comed. Visioscan	Cont. pores
Ser	+		+	+			+
Rt		+		+	+	+	+
Seb %	+		+	+			+
Cont. glan.	+	+	+	+			+
Comed. Vivascope		+			+	+	+
Comed. visioscan					+	+	+
Cont. pores	+	+	+	+	+	+	

**Table 2:** Correlation between the evaluated parameters on the chin region.

	Ser	Rt	Seb %	Cont. glan.	Comed. Vivascope	Comed. Visioscan	Cont. pores
Ser							
Rt			+	+	+	+	+
Seb %		+	+	+	+	+	+
Cont. glan.		+	+				
Comed. Vivascope		+	+				+
Comed. Visioscan		+	+				+
Cont. pores		+	+		+	+	

**Table 3:** Correlation between the evaluated parameters on the nose region.

	Ser	Rt	Seb %	Cont. glan.	Comed. Vivascope	Comed. Visioscan	Cont. pores
Ser		+	+			+	
Rt	+		+	+		+	
Seb %	+	+		+			
Cont. glan.		+	+				
Comed. Vivascope							+
Comed. Visioscan					+	+	+
Cont. pores					+	+	

According to the results obtained in the correlation study between the evaluated parameters, it was possible to observe that the sebum content in the infundibulum was positively correlated with the number of pores counted in the malar region, showing no correlation with the count of comedones. Another important aspect to consider is the correlation obtained from micro relief parameters with the number of pores in the malar region, as the same correlation was not observed in other regions.

The malar region is the region where the pores are most visible (greater presence of large pores), which are presented as “large valleys” in the images obtained by the Visioface^®^ VC 98 camera. For this parameter, a positive correlation was observed, thus, the analysis of the roughness and texture of the skin microrelief can be considered as an important aspect to evaluate in the pore size and quantity evaluation studies.

In the chin region, a positive correlation was observed between the sebum content, the roughness parameter (Rt), number of pores and comedones. The nose region did not present a positive correlation between the amount of sebum, number of active sebaceous glands, and the number of pores and comedones.

These different characteristics show that the skin is a complex structure and that several factors may be involved in the conformation of its characteristics and clinical aspects. Therefore, it is of great importance to obtain a broad and dynamic view of all the factors involved, and to evaluate them together, which will allow conclusive results to be obtained.

The present study also characterized structures such as pores and comedones by Reflectance Confocal Microscopy, which allows a thorough analysis of the different layers of the skin, as well as the structure of the hair follicle.

**FIGURE 6:**
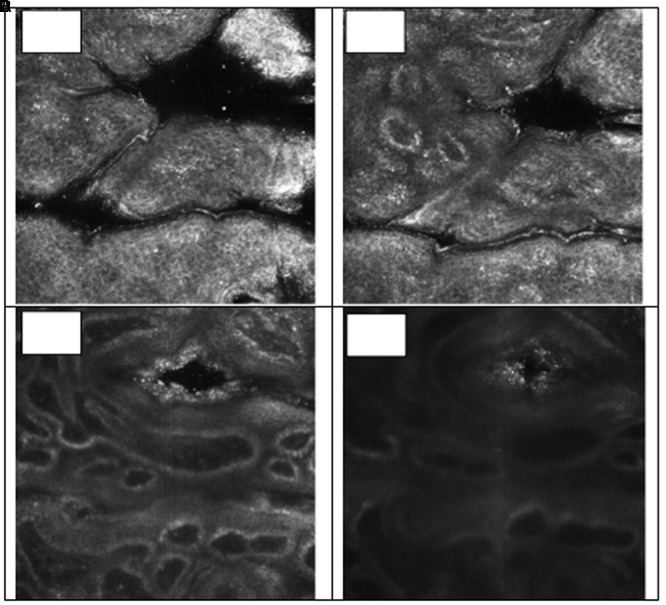
Visual aspect of pores in different skin depths 18 μm **(A)**, 36 μm **(B)**, 81 μm **(C)**, and 126 μm **(D)** – RCM.

**FIGURE 7:**
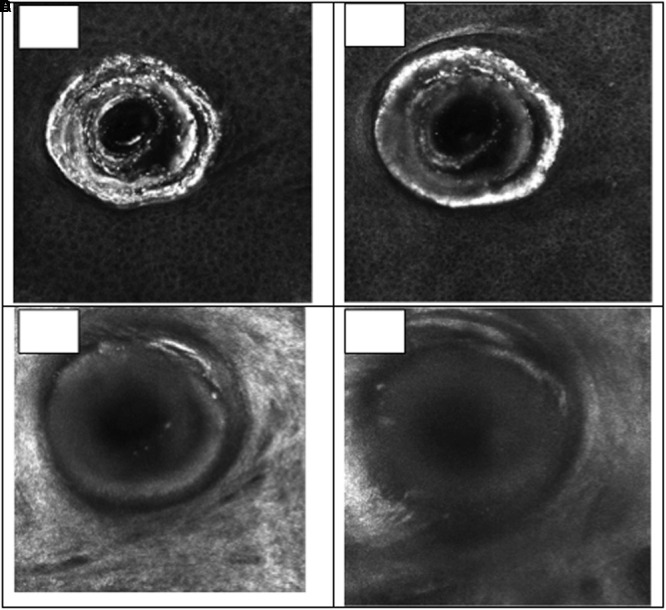
Visual aspect of an open comedone (chin region) in different skin depths 18 μm **(A)**, 36 μm **(B)**, 81 μm **(C)**, and 126 μm **(D)** – RCM.

**FIGURE 8:**
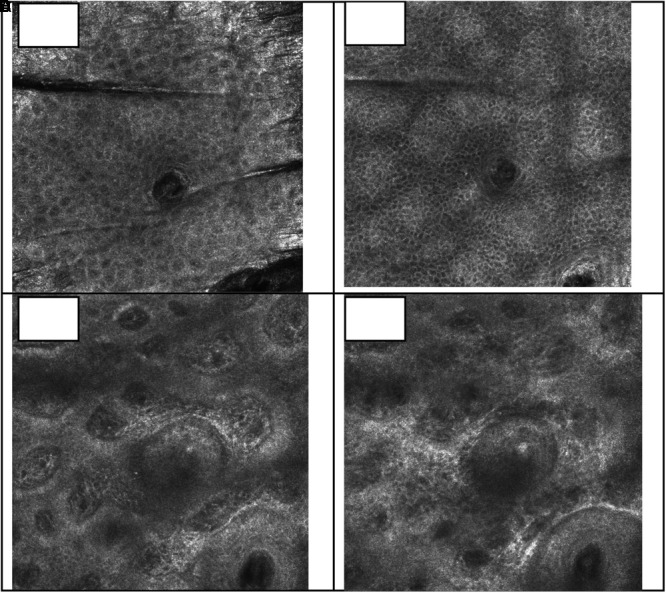
Visual aspect of a closed comedone (chin region) in different skin depths 18 μm **(A)**, 36 μm **(B)**, 81 μm **(C)**, and 126 μm **(D)** – RCM.

Pores are a depression in the stratum corneum layer, presenting a keratinized coating, represented by the white color, characteristic of keratin, and the lumen of the follicle has a larger diameter than a normal follicle (Figure 6). In the evaluation of the comedones, it was possible to observe the white mass which corresponds to a mixture of lipids and keratin, also presenting a pattern of hyperkeratinization, in the infundibulum cell layer (Figure 7). A normal closed comedone is presented in Figure 8, for comparison.

One of the crucial initial events for pore formation and acne lesions is hyperkeratinization of the follicle. The development of acne is related to the alteration of the structural physiology of the infundibulum, which is the region of the follicle that corresponds to the sebaceous gland outlet and to the external orifice of the follicle (Kurokawa et al., 2009). The regulation of the keratinization process is therefore, of great interest in order to prevent these undesirable clinical signs.

As the face presents specific regional characteristics, particularities such as its physiological and structural characteristics, may lead to differentiated clinical signs, showing the complexity of factors that influence the characteristics observed in the skin.

Finally, the different properties of skin that are related and which influence the appearance of undesirable clinical signs, evidence the necessity of a multifactorial approach for the effective improvement of oily skin in different facial regions (Jiang et al., 2018). The development of multifunctional cosmetic products that promote control of skin oils, regulate the keratinization process, improve micro relief and better structure the epidermis and dermis, will enable not only the improvement of oily skin conditions but will also improve or reduce the clinical signs that are a result of excessive oiliness, which are a major cause for concern and which results in a relentless search for cosmetic and dermatological products that address these issues (Wu et al., 2013; de Melo and Maia Campos, 2018).

## Conclusion

In the experimental conditions of this study, we can conclude that different face regions presented different characteristics related to oiliness and to the quantification and appearance of pores and comedones. In addition, sebaceous production, keratinization, microregulation and epidermal structure are all related to the clinical signs of oily skin, evidencing the need for the development of multifunctional products that act to improve oily skin conditions and its clinical signs.

## Author Contributions

PMC contributed in the elaboration of the study protocol, analysis and results discussion, and writing of the manuscript. DM contributed in the elaboration of the study protocol and experimental procedure, and analysis and results discussion. MM contributed to the results discussion and writing of the manuscript.

## Conflict of Interest Statement

The authors declare that the research was conducted in the absence of any commercial or financial relationships that could be construed as a potential conflict of interest.
