# Corrigendum: Use of Advanced Imaging Techniques for the Characterization of Oily Skin

**DOI:** 10.3389/fphys.2020.602226

**Published:** 2020-10-15

**Authors:** Patricia M. B. G. Maia Campos, Maisa O. Melo, Daiane G. Mercurio

**Affiliations:** School of Pharmaceutical Sciences of Ribeirão Preto, University of São Paulo, São Paulo, Brazil

**Keywords:** oily skin, biophysical and skin imaging techniques, acne, confocal microscopy, cosmetics

In the original article, we neglected to include the funder São Paulo Research Foundation—FAPESP, 2017/19278-0 to Patrícia Maria Berardo Gonçalves Maia Campos. The funding statement should read as “We would like to thank São Paulo Research Foundation—FAPESP (grant number 2017/19278-0) to PMC for financial support of our research.”

The authors apologize for this error and state that this does not change the scientific conclusions of the article in any way. The original article has been updated.

